# Establishment, authenticity, and characterization of cervical cancer cell lines

**DOI:** 10.1080/23723556.2022.2078628

**Published:** 2022-06-01

**Authors:** Ma de Lourdes Zuñiga Martinez, Carlos Miguel López Mendoza, Jared Tenorio Salazar, Alejandro Manuel García Carrancá, Marco Antonio Cerbón Cervantes, Luz Eugenia Alcántara-Quintana

**Affiliations:** a Posgrado en Ciencias Biomédicas Básicas, Universidad Autónoma de San Luis Potosí, San Luis Potosí, México; b Unidad de Innovación en Diagnóstico Celular y Molecular. Coordinación para la Innovación y la Aplicación de la Ciencia y Tecnología, San Luis Potosí, México; c Biotecnología, Unidad Periférica INCancerologíaDepartamento de Biología Molecular y , Ciudad de México, México; d – Facultad de Química, Universidad Nacional Autónoma de MéxicoUnidad de Investigación en Reproducción Humana, Instituto Nacional de Perinatología “Isidro Espinosa de los Reyes” , Ciudad de México, México; e Catedra CONACYT, Unidad de Innovación en Diagnóstico Celular y Molecular. Coordinación para la Innovación y la Aplicación de la Ciencia y Tecnología, San LuisPotosí, México

**Keywords:** Cell lines, characterization, authenticity, establishment, cervical cancer, *In vitro* models, HPV (human papilloma virus)

## Abstract

Cell lines have been considered excellent research models in many areas of biomedicine and, specifically, in the study of carcinogenesis. However, they cease to be effective models if their behavior changes. Although studies on the cross-contamination of cell lines originating from different tissues have been performed, little is known about cell lines derived from cervical neoplasia. We know that high-risk HPV (HR-HPV) is associated with the development of this type of cancer. This link between HPV infection and cancer was first established over 35 years ago when HPV16 DNA was found to be present in a large proportion of cervical cancer biopsies. The present review paper aims to report the status of the establishment, authenticity, and characterization of cervical cancer (CC) cell lines. This is a systematic review of articles on the establishment, authenticity, and characterization of CC cell lines, published from 1960 to date in the databases and in cell repository databases. 52 cell lines were identified in the literature. Only 25 cell lines were derived from cervical neoplasia, of which only 45.8% have a reported identity test (genomic fingerprint). Despite the increase in the establishment of cell lines of cervical neoplasia and the standards for the regulation of these study models, the criteria for their characterization continue to be diverse.

## Introduction

Even though cervical cancer (CC) may be preventable, it continues to occupy fourth place in the incidence of neoplasms worldwide, with 604,127 cases per year. In Mexico, it is the second most common cancer in the female population, with 9,439 cases per year.^1^ However, in middle and low-income countries, mortality can exceed 50% of cases. Strategies for prevention, timely diagnosis, and therapeutic interventions have been developed thanks to cancer research based on models using cell lines. The HPV life cycle is intrinsically linked to the programmed epithelial differentiation of the cervix. The majority of the focus has been on high-risk HPVs (HR-HPVs), which have been linked to cancer development.^2,3^ This link between HPV infection and cancer was first established over 35 years ago when HPV16 DNA was found to be present in a large proportion of cervical cancer biopsies.^4,5^ Cell lines are used in multiple areas of biomedicine, specifically in the study of carcinogenesis. A cell line is “an *in vitro* culture of cells that achieve indefinite survival and can be frozen and retrieved a theoretically infinite number of times.”^6^ When the right conditions are met, these cells will keep dividing and keep many of the characteristics of the cell type or tissue from which they came.^7^ The European Collection of Animal Cell Cultures, 2012,^8^ defines two types of cell lines according to the capacity of the cells to continue dividing; finite or senescent, which die after a fixed number of duplications, and continuous, which can be propagated indefinitely. Every cell line comes from a specific tissue or by cloning an already established line; each tissue has specific characteristics (morphology, organization, specialized functions, cell cycle, etc.). When a cell line is established, many of these characteristics are lost, such as cell-cell (or cell-tissue) junction, cell communication, and regulation.^9^ The characteristics of a cell line vary from one person to another. However, they all share certain features.

However, working with these models has a limited efficiency due to their behavior, i.e., this can change for two main reasons: contamination with other lines and misidentification.^10^ Multiple studies have demonstrated these issues, and it is estimated that between 18 and 36% of cell lines are contaminated or misidentified,^11^ with only 43% of cell lines considered “well identified.^12^ The cost of cell line contamination is high. For example, in the United States in 2013, investment in breast cancer research amounted to 370,000 million dollars, and it is estimated that at least 100 million dollars were lost in studies using cell lines that did not come from this tissue.^13^ In this case, not only are investment costs at stake, but also the credibility of the researchers and the inability to reproduce and transfer the results they come up with.

Dr. Gartler published the first report of contamination in cell lines in 1968,^14^ demonstrated by electrophoretic analysis of the enzymes Glucose 6 Phosphate Dehydrogenase (G6PD) and Phosphoglucomutase (PGM). He evaluated 30 different cell lines, finding that 18 of them, despite coming from the Caucasian race, presented the same polymorphism (A) as the HeLa cells, which come from the African-American race, concluding that it was contamination of these lines and introducing the concept of biochemical polymorphisms. Since then, multiple studies^15–20^corroborating his results have been conducted. Recently, the International Cell Line Authentication Committee (ICLAC) has reported 486 lines as misidentified, which has harmed at least 32,000 published articles.^21^

Capes-Davis et al. (2012) point out that a cell line is misidentified when its DNA profile is no longer consistent with that of the donor from which it was first obtained.^22^ Other researchers corroborate this fact since there is an error when describing cell lines according to the tissue of origin.^23–25^ For example, the ECV304 line is said to come from normal endothelial cells when in fact it comes from bladder cancer. The KB line is said to come from laryngeal carcinoma when in fact it is a clone of modified HeLa cells that have been grown in a lab. It’s also possible to mix up culture samples, cross-contaminate cell lines with each other, and get biological contamination from microorganisms like mycoplasma and fungi.^26^

Stacey (2007)^27^ indicates that there are three fundamental characteristics to ensure the quality of work with cell lines: 1) purity, that is, that they are free of microorganisms; 2) identity, which refers to the cells’ being who they say they are; and 3) stability, indicating that the genotype and phenotype must remain unchanged during growth and *in vitro* passages. Other criteria highlighted by cell culture researchers are the verification of viability, karyotyping, confirmation of the species of origin, specific cell identification (genomic fingerprinting), cell markers, genetic expressions, pluripotency (in the case of stem cell tissues), as well as quality controls in culture.^27^ Cell morphology (phenotypic changes) and ploidy (genotypic changes) are also things to keep an eye on in cell lines to make sure they have the right biological properties.^28^

The criteria for authentication and characterization of a line are very diverse among researchers. Therefore, international organizations such as the International Committee for Cell Line Authentication, the European Society for Animal Cell Technology, the World Biological Standards Institute, or the UK Cancer Research Coordinating Committee, among others, issue guidelines on the use, maintenance, development, and deposition of cell lines. Among the norms described by these organizations, the ethical aspects stand out, such as the need for the approval of the study by a research ethics committee, obtaining the informed consent of the donor, the transfer of rights to the biological material, etc. The data obtained on the tissue and the donor, the methods suggested to verify the authenticity of the cell line by DNA analysis, the criteria for its deposit in repositories, and even data on the establishment and characterization of the line should be included in the publications.^29,30^

Also, the techniques used in the characterization and authentication of cell lines are varied. For example, isoenzyme analysis determines the origin of the species of a given line and allows the detection of cross-contamination through the electrophoretic mobility of different isoenzymes. Cytogenetic analysis allows chromosomal markers to be identified.^31^ Genomic fingerprinting exploits the variability found in non-coding regions of the human genome, which is organized into repeated sequences (variable numbers of VNTR tandem repeats) of two types: minisatellites (10 to 100 bp) and microsatellites (2 to 5 bp), also called short tandem repeats (STRs). The analysis of these regions is necessary because the probability of two unrelated individuals having the same combination at a specific locus is less than 1%^9^ In addition, advances in technology have favored the evaluation of cell lines. From morphological observation of cells in culture by transmission or scanning electron microscopy, to the implementation of genomic analysis methods and molecular cytogenesis, researchers now have more accurate methods to assess the authenticity of cell lines and their growth characteristics in culture.

In summary, studies on cross-contamination of cell lines originating from diverse tissues have been performed,^32–36^ and the development of these biological models has increased significantly in recent decades. However, despite this, there is a lack of information on appropriately identified cervical neoplasia-derived cell lines. So, this review will show how cervical cancer (CC) cell lines are made, how they are authentic, and how they are characterized.

## Target

To show how cervical cancer (CC) cell lines have been set up, authenticated, and characterized over time.

## Materials and methods

The present study summarizes the current knowledge concerning the establishment and characterization of CC cell lines established between 1960 and 2020. It is a systematic review based on the phases of the Preferred Reporting Items for Systematic Reviews and Partial Analyses (PRISMA).^37^ The concepts and strategies for the systematic search to locate information from Pozos and Garrocho (2012) were also taken up.^38^

A search for publications concerning the establishment and characterization of CC cell lines from 1960 to 2020 was performed in the databases PubMed, Web of Science, Natura, SpringerLink, EBSCO, ScienceDirect, Ovid, Redalyc, PLOS, BMC, SciELO, PMC, Google, Google Scholar, BIG (Search for Global Information) and Academic Source. These cell repositories, including the American Type Culture Collection (ATCC), European Collection of Authenticated Cell Cultures (ECACC), Accegen, and Cellosaurus, were also looked at.

All titles that presented at least one of the following keywords: establishment, characterization, authenticity of cervical cancer cell lines were reviewed. Initially, papers that did not respond to any of the keywords, articles written in languages other than Spanish, English, or French, and titles without an abstract or text were eliminated. Next, abstracts of articles on the establishment and/or characterization of CC cell lines were obtained, excluding those in which the lines had originated from animal models, from other already established lines (i.e., by cloning, transfection, or genotypic modification), or those derived from Low-Grade Intraepithelial Lesions (LGEI).

Subsequently, references to CC lines were obtained and analyzed for their integration or exclusion in the study. The HeLa cell line was first ruled out because it had been reported as a contaminant of other cell lines and because no specific papers on how it was made or how it was characterized were found. However, many papers on some of its characteristics were found.

The documents were then obtained in PDF format, and the data was organized under two headings: 1) the establishment of CC cell lines and 2) the characterization of the lines. An EXCEL database was constructed considering the following: year of publication, authors, journal, names of the established cell lines, general data (age, race, histopathological diagnosis, tissue origin of the sample, previous treatment), characterization methods (morphological, growth characteristics, cell population, doubling time, contact inhibition, adhesion, saturation density, karyotype, HPV genotyping, isoenzymatic analysis, genomic fingerprinting), and ethical aspects.

**Figure 1.**
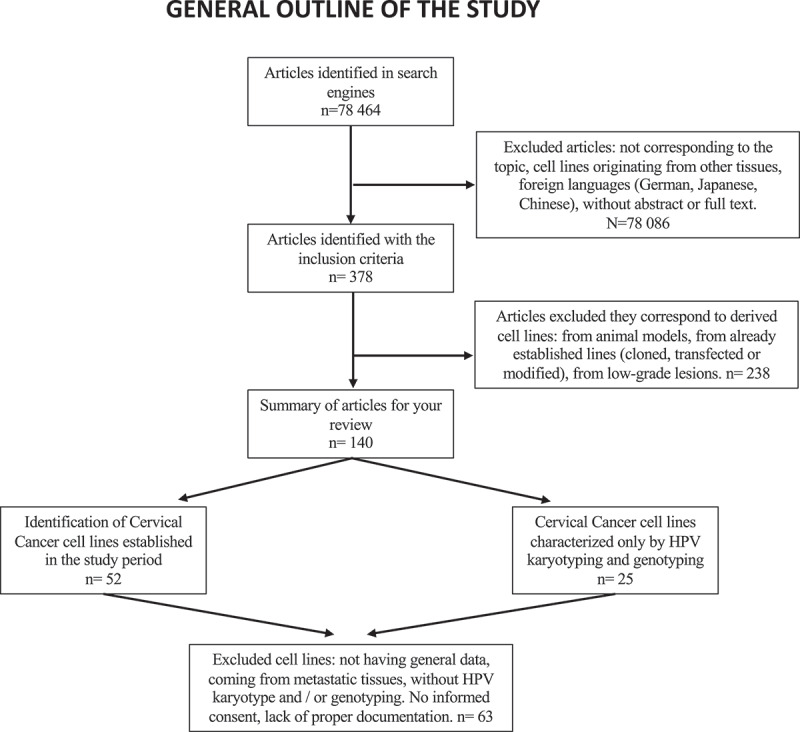
General outline of the study.

To verify that the lines were derived from cervical neoplasia, they were examined for: a) karyotype, b) HPV genotyping (due to its association with cervical carcinogenesis), and c) tissue of origin. A second document review of each line was performed to identify missing data regarding these three conditions. The data on the methods used to verify the authenticity and characterization of each line were subsequently organized into tables (Figure 1). Finally, the results, according to their classification, were expressed in percentages.

## Results

A total of 378 abstracts were reviewed from which 140 scientific articles were obtained. In these papers, the establishment of 52 CC cell lines from 1960 to 2020 was reported.

In terms of origin, we found that the countries reporting the highest percentage (53.7%) of established lines are in Asia, followed by North America (24.1%) and Latin America (11.1%), while European countries report the lowest percentage (9.3%) of established lines. Only one of the lines (DoTc2) did not report this data. Table 1 shows data that was consistent across all reviewed papers on the establishment and characterization of CC cell lines in the study period. Only 48% of the 52 cell lines were identified as derived from cervical neoplasia as they come directly from cervical tissue and have karyotyping and HPV genotyping reported as methods to assess, respectively, authenticity and determine the main characteristics of the line. The remaining lines (52%) lack proper identification because: (a) they are derived from metastatic tissues (lymph nodes-HT3, MS751-, intestinal epithelium-CaSki-, omentum-ME180-) or cells derived from fluids such as ascitic fluid (EC82, SFCC, SKS), b) no karyotype report (EC82), no genotyping (SFCC), or c) no general data report (age).

**Table 1.** Cervical cell lines established between 1960–2020

Criteria	No. cell line identified	Percentage
Not reported karyotype	13	25.0
Not reported genotyping HPV	3	5.8
Ciudad de México	7	13.5
Not reported general date or karyotype/genotyping	4	7.7
Karyotyping + Genotyping HPV	25	48.0
Total	52	100

According to Table 2, which contains the data of the 25 CC lines identified according to their racial origin, the following results were obtained: 10 of them (40%) come from Caucasian women, 13 (52%) from Asian women, and only 2 (8%) from Latin American women. Many lines (40%) correspond to women between 30 and 39 years of age, followed by those obtained from women between 40 and 49 years of age (28%) and those between 60 and 69 years of age (20%). A few lines (8%) derive from women in their 50s and just one line does not report the data (INBL).

**Table 2.** Cervical cancer cell lines characterized by karyotype and genotyping HPV

No.	Cell line	Histology diagnosis	Race	Age	HPV	Karyotype	Reference
1	C4	Exophytic invasive squamous carcinoma of the cervix stage II, grade IV	C	41	HPV18	Hyperdiploid (45)	Auersperg^39–41^
2	C33	Invasive cervical carcinoma grade IV	C	66	Negative for HPV	Hyperdiploid (45)	Auersperg,^39–41^
3	SiHa	Squamous cell carcinoma stage II	A	55	HPV16	Hypertriploid (69)	Friedl^42^
4	SW756	Poorly differentiated invasive squamous carcinoma of the uterine cervix	C	46	HPV18	Triploid (76)	Freedman;^43^ Popescu^44^
5	SKGIII	Uterine cervical cancer stage II	A	38	HPV16	Aneuploidy (42)	Nozawa;^45^ Shirasawa^46^
6	HX151c	Poorly differentiated squamous cell carcinoma stage IB	C	30	HPV16	Aneuploidy (71)	Kelland;^47^ Spencer^48^
7	HX155c	Moderately differentiated squamous cell carcinoma stage IB	C	44	HPV16	Aneuploidy (74)	Kelland;^47^ Spencer^48^
8	HX156c	Poorly differentiated squamous cell carcinoma stage IIB	C	31	HPV16	Aneuploidy (74)	Kelland;^47^ Spencer^48^
9	HX160c	Moderately well-differentiated squamous cell carcinoma stage IB	C	44	HPV16	Aneuploidy (73)	Kelland;^47^ Spencer^48^
10	XH1	Invasive focally keratinizing adenosquamous	C	32	HPV16	Aneuploidy (78)	Han^49^
11	CaLo	Epidermoid cervical carcinoma of keratinized large cell from nonmetastatic tumor stage IIB	L	55	HPV18	Aneuploidy (50)	Monroy^50^
12	INBL	Epidermoid cervical carcinoma of keratinized large cell from metastatic tumor stage IVB	L	NR	HPV18	Aneuploidy (50)	Monroy^50^
13	CUMC-3	Invasive nonkeratinizing squamous cell carcinoma stage IIB	A	32	HPV16	Hypotetraploid (78)	Kim^51^
14	CUMC-6	Squamous cell carcinoma of the cervix uteri	A	31	HPV16-18	Diploid/trisomy (47)	Kim^52^
15	TC-140	Moderately differentiated squamous cell carcinoma with metastases to lymph nodes	C	39	HPV16	Triploid (61)	Braun;^53^ Mark^54^
16	TC-146	Moderately differentiated epidermoid carcinoma *in situ* of the cervix	C	36	HPV16	Triploid (48)	Mark^54^
17	SiSo	Adenocarcinoma of the uterine cervix stage IB	A	67	HPV18	Aneuploid (61)	Sonoda^55^
18	CX	Squamous cell carcinoma of the uterine cervix stage IIA	A	48	Negative for HPV	Euploidy	Chou^56^
19	CA	Non-keratinizing squamous cell carcinoma stage IB2	A	36	Negative for HPV	Aneuploid (65)	Isaka^57^
20	HHUS	Uterine cervical keratinizing squamous cell carcinoma	A	64	HPV59	Diploid (48)	Ishiwata^58^
21	RSBS-9	Moderately differentiated keratinizing squamous cell carcinoma stage III	A	49	HPV16	Aneuploid (53)	Javed^59^
22	RSBS-14	Moderately differentiated non-keratinizing squamous cell carcinoma stage III	A	34	HPV16	Aneuploid (47)	Javed^59^
23	RSBS-23	Poorly differentiated non-keratinizing squamous cell carcinoma stage III	A	45	HPV16	Aneuploid (56)	Javed^59^
24	RSBS-43	Moderately differentiated adenocarcinoma of uterine cervix stage III	A	63	HPV16	Aneuploid (51)	Javed^59^
25	HUUCLEC	Human uterine cervical lymphoepithelial carcinoma	A	61	HPV56	Aneuploid (56)	Kiguchi^60^

C= Caucasian, A= Asian, L= Latin American, NR= Not reported data.

Regarding the type of neoplasm, 76% of the lines are described as epithelial carcinomas and only 12% as adenocarcinomas (×H1, SiSo, RSBS-43), 8% of the authors do not specify whether it is squamous or glandular (CA and SKG-III), and only one line (4%) is said to come from a lymphoepithelial type cancer (HUUCLE) determined by histology. Concerning the degree of progression of the neoplasm, we found great variability among the authors since the majority (80%) only refer to the stage according to the FIGO classification and only 4% include the grade, while 16% do not refer to the progression of cancer. The lines classified as stage I occupied 28% of the total, those included in stages II and III were 20%, and only 16% came from metastatic cervical neoplasms (stage IV).

Because of the association of cervical cancer with high-risk HPVs, viral genotyping has become an important element in cell line specificity. Of particular interest for study in carcinogenesis are those that have been reported: a) positive for viral load and b) infected with more than one HPV type most of the lines (56%) are described as HPV16, followed by those characterized as HPV18 (C4, SW756, CaLo, INBL, SiSo) and HPV negative (C33, CX, CA). Only one line was reported as HPV59 (HHUS) and one as HPV56 (HUUCLEC). In the line named CUMC-6, both type 16 and type 18 viral genomes were identified.

All lines are tumorigenic, so the karyotypes showed aberrations in different chromosomes and were referred to as aneuploid (60%), triploid (12%), diploid or hyperdiploid (8%), respectively. A few have been described with other chromosomal alterations, such as SiHa (hypertriploid), CUMC-3 (hypotetraploid), and, most notably, CX, which was reported as euploid (46 chromosomes), despite structural alterations in the XX chromosome.

Table 3 indicates the data reported for the characterization of each line; these include those concerning 1) culture conditions (cell division time, growth density, contact inhibition), 2) confirmation of line identity (genomic fingerprinting, isoenzyme analysis, confirmation of tissue of origin) and 3) confirmation of non-contamination.

**Table 3.** Methods to evaluate the authenticity and characterization of cervical cell lines

No.	Cell line	T_D_	No. Passages	Contact inhibition	Saturation density	Tumorigenicity	Uniqueness	Isoenzyme analysis	Contamination	Confirmation the tissue of origin
1	C4	24-48h	90	No	NR	Collagen gel, hamster model	SRT, transcriptome	G6PD-B	Free of HeLa	Cytology, histochemistry,
2	C33	24-76h	70	No	NR	Murine model	SRT, transcriptome	G6PD-B, PGM1–1, AK-1	Free of HeLa	NR
3	SiHa	2.6 days	NR	NR	NR	Murine model	SRT, transcriptome	G6PD-B, PGM1–1, AK-1	Free of mycoplasma, or HeLa	Desmosomes, tonofilaments
4	SW756	1.6 days	100	NR	NR	Murine model	SRT, transcriptome	G6PD-B, GLO-1,2	Free of mycoplasma, HeLa, ME180, or C4 I	HLA typing (A1, 24, B8, 44: Cw2, x; D2 W2, 6Y)
5	SKG-III	60-67h	80	No	4x10^5^ cell/cm^2^	Murine model	SRT, transcriptome	G6PD-B	Free of HeLa	Desmosomes, tonofilaments, HLA-A 2402
6	HX151c	42h	70	NR	Low	Murine model	NR	G6PDB. PGM1–1, PGM3-a, ACP1-BA, PGD-A	Free of mycoplasma, or HeLa	Vimentin, cytokeratin
7	HX155c	48h	50	NR	Low	Murine model	NR	G6PDB. PGM1–1, PGM3-a, ACP1-B, PGD-A	Free of mycoplasma, or HeLa	Vimentin, cytokeratin
8	HX156c	30h	75	NR	High	Murine model	NR	G6PDB. PGM1–1, PGM3-b, ACP1-BA, PGD-A	Free of mycoplasma, or HeLa	Cytokeratin
9	HX160c	68h	50	NR	Low	Murine model	NR	G6PDB. PGM1–1, PGM3-b, ACP1-B, PGD-A	Free of mycoplasma, or HeLa	Vimentin, cytokeratin
10	XH1	16.2h	100	NR	NR	Murine model	SRT	MS1	Free of mycoplasma, HeLa, A431, CaSki, and Bowes melanoma	Antibodies to membrane antigen, carcinoembryonic antigen, desmin, vimentin and cytokeratin
11	CaLo	3–4 days	50	No	High (>15,000 cell/ml)	NR	NR	NR	NR	Desmogeín-1
12	INBL	3–4 days	50	No	High (>15,000 cell/ml)	NR	NR	NR	NR	Desmogeín-1, HLA-A11
13	CUMC-3	48h	310	No	NR	Murine model	NR	G6PD-B, LDH-4, PGM	Free of HeLa	Desmosomes, tonofilaments, HLA-DRB1 0401
14	CUMC-6	36h	300	No	NR	Murine model with lung metastases	NR	G6PD-B, LDH, PGM	Free of HeLa	Desmosomes, tonofilaments, HLA-DQw3
15	TC-140	NR	NR	No	1x10^5^ cell/cm^2^	Murine model	NR	NR	Free of mycoplasma	Histology
16	TC-146	NR	NR	No	1x10^5^ cell/cm^2^	Murine model	FISH	NR	Free of mycoplasma	Histology
17	SiSo	24-35h	34	No	4x10^5^ cell/cm^2^	Murine model	STR, transcriptome	NR	NR	Mucin, cytokeratin, epithelial membrane antigen
18	CX	20h	NR	NR	NR	Not tumorigenic	RPPA, SNP, transcriptome	NR	Free of mycoplasma	Cytokeratin, desmosomes
19	CA	14.3h	280	No	NR	Murine model	SRT	NR	NR	Carcinoembryonic antigen, antígeno polipéptido de tejido
20	HHUS	67h	70	No	3.9x10^4^ cell/cm^2^	Murine model	NR	NR	NR	Desmosomes, tonofilaments, carcinoembryonic antigen, cytokeratin
21	RSBS-9	48h	NR	Yes	NR	Tumorosphere	VNTR	NR	Free of mycoplasma	Epithelial membrane antigen, cytokeratin
22	RSBS-14	48h	NR	Yes	NR	Tumorosphere	VNTR	NR	Free of mycoplasma	Epithelial membrane antigen, cytokeratin
23	RSBS-23	48h	NR	Yes	NR	Tumorosphere	VNTR	NR	Free of mycoplasma	Epithelial membrane antigen, cytokeratin
24	RSBS-43	48h	NR	Yes	NR	Tumorosphere	VNTR	NR	Free of mycoplasma	Epithelial membrane antigen, cytokeratin
25	HUUCLEC	70h	60	No	NR	Not tumorigenic	NR	NR	Free of virus Epstein y Barr	Cytokeratin, epithelial membrane antigen, carcinoembryonic antigen

T_D_: Doubling time, NR= Not reported data, STR: Short Tandem Repeats, SNP: single-nucleotide polymorphisms, VNTR: variable number of tandem repeats, MS31: Hypervariable minisatellite probing, FISH: fluorescence *in situ* hybridization, RPPA: reverse phase protein arrays, ACP-1: human red cell acid phosphatase, AK-1: adenylate kinase polymorphism, G6PD: glucose 6 phosphate dehydrogenase, PGM: phosphoglucomutase, HLA: human leukocyte antigen

Although many of the data reported in the studies were in general, some of the most constant ones were the time in which the cell population in culture doubles its population, as well as the confirmation of the tissue of origin. However, a considerable percentage (24%) of the total lines report 5 or fewer characteristics (some only two-TC140-); the majority (68%) include 6 to 7 criteria in their characterization (including authenticity and free of microorganisms), while only 8% include 8 to 9 (SKG-III and SiSo, respectively).

Among the lines with accelerated growth (24 h) were XH1, CX, and CA, followed by those with intermediate growth (24–48 h), which corresponded to 48%, while 24% of the lines presented slow growth times (48–76 h). Two lines (8%) - CaLo, INBL-were reported with the slowest cell division times (more than 76 h) and two others (TC140, TC146) did not report the data. The number of times cells were subcultured varied from line to line, from 34 passages (SiSo) to 300 (CUMC-6); only a few lines (SiHa, CX, TC140, TC146, RSBS9, RSBS14, RSBS23, RSBS43) do not have the report.

One of the criteria rarely found in the publications was the inhibition of cell growth by contact. Only in 11 lines (44%) was this feature described; in seven of them their cells do not stop dividing upon contact with others, as happens in neoplastic tissues, while in four lines (RSBS-9, RSBS-14, RSBS-23, and RSBS-43) contact inhibition was documented. Regarding the density at which the cells grow, in 36% (9 cell lines), this characteristic has been reported using qualitative (high, low) and quantitative (various) scales as shown in the table.

On the other hand, the characterization of tumorigenic capacity was established in 21 lines (84%); the privileged model for this was murine (18 lines), followed by 3D cultures (4 lines), while in three cell lines (CaLo, INBL, and CX) no publications were found; only one line (HUUCLEC) reported the absence of carcinogenesis in a murine model after inoculation. Regarding the establishment of line specificity and identity, methods based on DNA analysis (SRT, VNTR, SNP, FISH) were recurrent (52%) among the investigators (13 lines), whether used alone or in combination with RNA analysis (transcriptome) or protein expression (proteome). However, in only 44% of the lines, no other genetic tests were done other than karyotyping.

Likewise, the isoenzymatic analysis of biochemical polymorphisms seems to be a common criterion for the lines established before the 1990s, since in 11 lines (44%), established in that period, this data was documented, while for the rest it was not; the most studied enzymatic polymorphisms were in G6PD, PGM1 and 3, AK-1 and ACP1. The only line that didn’t report on this was C33. All the other lines reported on either a) specific epithelial tissue antigens (vimentin, desmogein, epithelial membrane antigen, etc.), specific neoplastic tissue antigens (carcinoembryonic antigen), histochemical or morphological studies, or a combination of these.

On the contrary, and even though the problem of cross-contamination between cell lines, as well as contamination by biological organisms, has been exposed since 1968, in nine lines (36%) no evidence was found for the absence of intra-or interspecies contamination, in 12 lines (48%) only contamination with mycoplasma or with another microorganism (Epstein-Barr virus) was evaluated, in three (12%) the absence of contamination with HeLa cells was reported, and only in one line (SW756) contamination with mycoplasma and HeLa cells were evaluated together.

Finally, in 76% of the lines identified, no ethical aspects were mentioned although the inclusion of these aspects has become an indispensable requirement in the publication of studies on the establishment of cell lines, in the procurement and use of tissues, as well as in the manipulation of genetic material. Likewise, ethical criteria are particularly important concerning the procurement of biological tissues, the transfer of rights, and the approval of the study by an ethics committee.

## Discussion

### Establishment of cervical cancer cell lines

78.8% of cervical cancer cell lines reported between 1960 and 2020 emerged from developed countries, such as Canada, the USA, Japan, the United Kingdom, and South Korea. A likely consequence of its high investment in research and development (R & D), however, is that the USA is the nation that invests the most in this concept (28.1% of national gross expenditure), followed by the European Union (19%) and Japan (10%). The remaining lines (21.2%) were developed in countries considered in transition (Mexico, Brazil, Thailand), where R & D investment is not so strong.^61^

However, only 48% of the lines that were reported came from cervical tissue, so they can be used as biological models of how malignant cells behave in real life. Culturist researchers say that it is important to know where the cell line came from so that future research can show how the system model works.^62,63^

Different cervical cancer cell lines are important because they allow us to know how the cells will react to a stimulus or treatment when they are grown in xenograft cultures in vivo. This is because we need to know how the cells will react to a stimulus or treatment when they are grown in xenograft cultures.

In another respect, 9 of 25 cervical cancer cell lines were established between 1960 and 1990, so the obtaining of the cells was understood as an extension of the diagnostic process in which the patient did not need to be consulted.^64^ According to international organizations, only one cell line had its origin confirmed by the donor.^65^ At least 12 cell lines, established after the 1990s, lack an ethical review, i.e., obtaining biological material and consent or rights from the donor. In this case, if an ethics committee had given its approval to the methods for getting the tissue, that would be something that is not talked about.

### Authentication of cervical cancer cell lines

The American National Standards Institute (ANSI) and the American Type Culture Collection (ATCC) and other international agencies (UKCCR, ECACC) promote the use of genomic analysis techniques (STRs, VNTS, minisatellites, etc.) for identity verification of human cell lines (ASN-0002) that are unique and unrepeatable. STR analysis is the most common method used by the authors. It is a standard method that is fast, cheap, and has been tested by cell banks. The data is also consistent.^22,66^

Other techniques that corroborate the uniqueness of cell lines are HPV integration sites, microsatellite stability, HLA typing, polymorphisms, as well as mutations and gene expression. In our study, few researchers reported these latest data. One disadvantage of this is that some cell lines tend to undergo genetic changes with continuous passages in culture. In malignant cells, there may be loss of heterozygosity and increased instability of microsatellites.^67,68^

Also, persistent high-risk papillomavirus infection, i.e., HVP16, has been significantly associated with neoplastic progression. However, the determinants of viral persistence and clearance are not yet well understood. Many studies have been done to compare the responses of different types of CC cell lines to the administration of antineoplastic substances, but the most common models have been genotyped with HPV16 or 18. This means that little is known about how neoplastic cells react to another or no viral type.

In recent studies conducted in Europe, North America, and Latin America, in more than 1000 women with positive samples for HPV, they show the presence of multiple infections at high and low risk in more than 30% of the positive cases studied.^69–73^ Multiple infections appear to be more common in women under the age of 30, whereas viral type 16 excels in single and multiple infections associated with high-and low-risk HPV. A single viral genotype has been detected in a single sample.^66^ Some studies have shown that having a lot of HPV can make it more likely that you’ll get high-grade lesions and get cancer, but they aren’t sure yet.

In our review, a single cell line reports the viral presence of both HPV16 and 18 in a woman over 30 years of Asian origin. The development of models with these characteristics could help us learn more about how many infections affect cervical carcinogenesis, which would help us figure out who is at risk in groups.

At least five lines of cervical cancer do not report the data and, although few studies on cell line contamination include these five lines of cervical cancer,^15,20,74,75^ their data are not conclusive if these lines were found or not contaminated; the International Committee for Authentication of Cell Lines,^65^ reports five lines of CC that have been found as contaminants of other-HeLa, ME180, SKGII, C33A, and TCO2–. The above data shows that it’s important to report how to keep cultures healthy and cell lines true.

### Characterization of cervical cancer cell lines

Knowing the morphological characteristics of cells in culture allows identifying changes in response to different environmental conditions (i.e., changes in the substrate, cryopreservation, cell density, phenotypic and genotypic changes, etc.) that require constant monitoring to prevent the invalidation of research work.^7,32^

Normal cells usually stop dividing at a high cell density. They block in the G_1_ phase of the cell cycle and deteriorate very little. Tumor cells result in morphological deterioration but continue to proliferate beyond confluence. Freshner^32^ suggests expressing cell density in cell/cm2 to avoid ambiguities in interpretations such as high, medium, and low of the correct seeding density and subculture interval is done by performing a growth curve to establish the deviations from this pattern and prevent cell deterioration.

Likewise, chromosome content in cancer lines is aneuploid (abnormal chromosome content) and heteroploid (variability in chromosome number in the same cell population) as a result of alteration of tumor suppressor proteins, i.e., p53 and Rb, leading to genetic instability throughout the subcultures.^76^ Viability and karyotype have also been used to check the genetic stability of cell cultures and to see if cells have changed.^77^

Phenotypic changes can occur during different passages of the cultures or by intra-and interspecies contamination, so it is important to report the number of passages that have had the established line, as well as the purity tests used in the laboratory. Karyotype is the most commonly used method to monitor the genetic instability of culture and determine the malignancy of the cell line, yet almost half of the identified lines do not report it.

Ultimately, the results of the study indicate a significant increase in the establishment of cervical cancer cell lines in recent decades. However, despite the regulations of international agencies, authentication and characterization criteria remain very diverse among researchers and some dates, i.e., ethical review, are even unaffordable for the majority.

## Data Availability

The authors hereby confirm that the data supporting the conclusions of this study are available in supplementary material.
